# Determination of lithium in human serum by isotope dilution atomic absorption spectrometry

**DOI:** 10.1007/s00216-021-03636-6

**Published:** 2021-09-10

**Authors:** Alexander Winckelmann, Dalia Morcillo, Silke Richter, Sebastian Recknagel, Jens Riedel, Jochen Vogl, Ulrich Panne, Carlos Abad

**Affiliations:** 1 Department of Chemistry, Humboldt-Universität zu Berlin, Brook-Taylor-Str. 2, 12489 Berlin, Germany; 2 Bundesanstalt für Materialforschung und –prüfung (BAM), Richard-Willstätter-Str. 11, 12489 Berlin, Germany

**Keywords:** Lithium, Human serum, Isotope dilution, Atomic absorption spectrometry, High-resolution continuum source graphite furnace atomic absorption spectrometry

## Abstract

The therapeutic dose of lithium (Li) compounds, which are widely used for the treatment of psychiatric and hematologic disorders, is close to its toxic level; therefore, drug monitoring protocols are mandatory. Herein, we propose a fast, simple, and low-cost analytical procedure for the traceable determination of Li concentration in human serum, based on the monitoring of the Li isotope dilution through the partially resolved isotope shift in its electronic transition around 670.80 nm using a commercially available high-resolution continuum source graphite furnace atomic absorption spectrometer. With this technique, serum samples only require acidic digestion before analysis. The procedure requires three measurements—an enriched ^6^Li spike, a mixture of a certified standard solution and spike, and a mixture of the sample and spike with a nominal ^7^Li/^6^Li ratio of 0.82. Lanthanum has been used as an internal spectral standard for wavelength correction. The spectra are described as the linear superposition of the contributions of the respective isotopes, each consisting of a spin-orbit doublet, which can be expressed as Gaussian components with constant spectral position and width and different relative intensity, reflecting the isotope ratio in the sample. Both the spectral constants and the correlation between isotope ratio and relative band intensity have been experimentally obtained using commercially available materials enriched with Li isotopes. The Li characteristic mass (*m*_*c*_) obtained corresponds to 0.6 pg. The procedure has been validated using five human serum certified reference materials. The results are metrologically comparable and compatible to the certified values. The measurement uncertainties are comparable to those obtained by the more complex and expensive technique, isotope dilution mass spectrometry.

**Graphical abstract:**

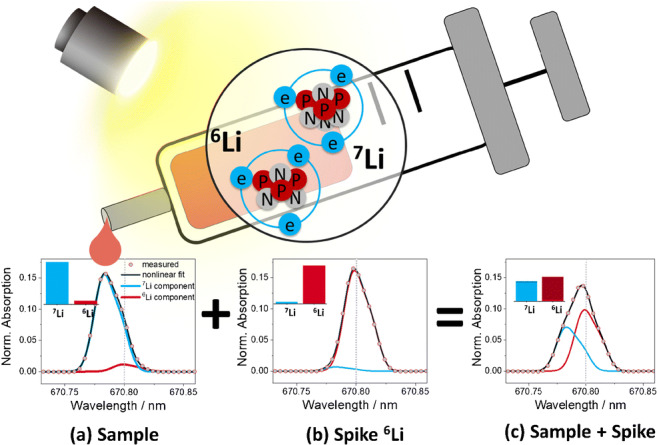

**Supplementary Information:**

The online version contains supplementary material available at 10.1007/s00216-021-03636-6.

## Introduction

The therapeutic effects of lithium (Li) on the brain and blood are well known and applied for the treatment of manic depression, granulocytopenia generated by radiation and chemotherapy, and immunoglobulin stimulation. Li has a narrow therapeutic range of 0.6–1.0 mmol L^−1^. Concentrations of Li above 1.5 mmol L^−1^ in serum can be toxic [1]; hence, close monitoring of the Li concentration is mandatory to ensure effective and secure treatment. Considering the interindividual variation of the half-life of Li in patients, a quick and accurate analysis is required [2]. Currently, the primary analytical techniques used for Li quantification are based on relative measurements, which require reference serum standards for calibration, thereby hampering traceability and comparability.

Several analytical methods for the quantification of Li in biological samples have been proposed, including atomic absorption spectrophotometry (AAS) [3], inductively coupled plasma (ICP) optical emission spectroscopy (OES) [4], and ICP mass spectrometry (MS) [5]. The development of ICP-based instrumentation has facilitated the simultaneous quantification of several elements of medical interest with sufficient accuracy (trueness and precision) [6–8]. However, ICP-based methods generally require laborious sample preparation, and are expensive and matrix dependent. In this context, methodologies like isotope dilution (ID) are less matrix sensitive and provide reduced uncertainties in the measurement [9, 10]. ID is an absolute technique that improves metrological quality and provides an almost standard-free calibration approach, once the isotope spike is characterized.

Quantification of Li by ID-ICP-MS has been proposed for geological and biological samples [11, 12]. ID-MS can provide analyte concentrations that are traceable to the international system of units (SI) and thus enabling comparability, when certain requirements are fulfilled [13]. However, in the case of Li, MS-based methods are prone to large mass bias and matrix effects and huge instrumental drift, which is primarily due to a low mass-to-charge ratio and the resulting high relative mass difference between the two Li isotopes [14]. The high extent of the mass bias and its high fluctuation hinder its accurate correction, which in turn negatively affects the measurement uncertainty.

Some attractive alternatives to ID-MS techniques are based on the isotope shift in atomic and molecular electronic spectra. For example, ID-AAS was proposed by Brost et al. to monitor the absorption coefficient of naturally occurring Li in human plasma and enriched isotopic materials using natural and ^6^Li-enriched hollow cathode lamps. The bias for Li recovery in plasma ranged from −2.8 to 0.6% [15]. However, the availability of isotopically enriched hollow cathode lamps limits the practical application of this method.

In recent years, optical spectrometry has flourished for isotope analysis. The use of a continuum light source coupled with a high-resolution echelle spectrometer and a charge-coupled device detector allows the monitoring of the electronic transition of transient diatomic molecules. The isotope shift in the electronic spectra of these diatomic molecules depends on the reduced mass and is larger than those observed in atomic spectra [16]. Thus, the monitoring of the isotope shift and the relative intensities of the isotopologue couple Al^35^Cl/Al^37^Cl proved useful for the trace analysis of Cl via ID using a commercially available high-resolution continuum source graphite furnace molecular absorption spectrometer (HR-CS-GF-MAS) [17]. The same approach was applied for the trace analysis of Br and Ca [18, 19]. Additionally, accurate isotope ratios, which are the core of ID-MS applications, can be achieved by HR-CS-MAS and HR-CS-AAS using modern methods for data analysis like partial least square regression and machine learning, as was demonstrated for B and Li, respectively [20, 21]. In this latter work on Li isotope ratio analysis, the electronic transition 2^2^P ← 2^2^S was explored. This transition can be used for Li quantification by ID.

This work investigates the determination of the amount of Li in human serum reference materials based on an ID approach applied to HR-CS-GFAAS. Since the atomic spectra of Li follow a nonlinear Gaussian function, this can be used for isotope ratio analysis for SI traceability instead of a calibration model by machine learning. The performance achieved by ID-HR-GFAAS in the present study is compared with those obtained by certificates of analysis of reference materials and previous reports on ID-MS.

## Materials and methods

### Sample preparation

High-purity deionized water with a resistivity of 18 MΩ cm obtained from a Milli-Q system (Millipore gradient, Merck Millipore, Darmstadt, Germany) was used throughout the experiments. Nitric acid (HNO_3_; EMSURE®, Merck, Darmstadt, Germany) was used after purification by subboiling distillation in PFA containers. The samples analyzed consisted of five serum certified reference materials (CRMs): BCR 304 (Joint Research Centre, Belgium), ERM-DA250a and ERM-DA251a (LGC Limited, UK), Seronorm L-1 (SERO AS, Norway), and Seronorm L-2 (SERO AS, Norway). A spike solution was prepared from metallic ^6^Li (≈ 95% ^6^Li) in 2% HNO_3_ with a mass concentration of around 0.5 mg L^−1^. For the ID analysis, 0.3 g of each serum sample was digested in triplicate using 2 mL HNO_3_ (35%) and 1 mL 15% H_2_O_2_ (Suprapur®, Merck, Darmstadt, Germany) at 100 °C for 30 min. After digestion, 0.7 g of spike solution was added, and the solution was filled up to 25 mL with 2% HNO_3_. For reverse ID [9], 0.3 g of an ICP standard solution (traceable to NIST SRM® 3129a, Certipur®, Merck, Darmstadt, Germany) was mixed with 0.7 g of the spike solution and filled up to 10 mL with 2% HNO_3_. An ^7^Li-enriched solution was used for optimization of the fit parameters and it was prepared by diluting ^7^Li_2_CO_3_ (≥99 atom%, 99% ^7^Li, Sigma-Aldrich, USA) in 2% HNO_3_ for a mass concentration of around 0.5 mg L^−1^.

### Atomic absorption spectrometry measurements

A ContrAA 800D HR-CS-GFAAS model (Analytik Jena, Germany) with a graphite furnace (PIN platform) was used for all measurements. The wavelength of the instrument was centered at 670.7845 nm. The optimized measurement conditions provided in our recent work were applied [21]. However, owing to the low vapor pressure of La, the atomization temperature was increased to 2500 °C. For each measurement, 10 μL of digested sample was injected. The Li concentration of the injected solutions was adjusted to match an extinction of 3.0 integrated from 670.7361 to 670.8680 nm. A La ICP standard solution (Certipur®, Merck, Darmstadt, Germany) was added as an internal spectral standard with a final mass concentration of 2 g L^−1^. Each sample was measured ten times, and 150 spectra were recorded during each atomization. The same procedure was applied for the ^6^Li spike solution and the spiked standard solution (used for reverse ID) and for the ^7^Li solution. Between each sample, a blank sample was measured five times.

### Data analysis

For each measurement, 150 spectra were collected during the Li atomization and averaged. Spectral data were preprocessed and reduced using the MATLAB software (R2020a, The MathWorks Inc., USA). The spectral data of each measurement were compiled, transformed, and integrated from three-dimensional to two-dimensional spectra by converting them to their average. Finally, the area of the Li line was normalized to the unity. Using the *fminsearch* function in MATLAB, the spectra were fit to Eq. 1, a sum of four Gaussian functions, where *c*_*i*_ is the central wavelength, *w*_*i*_ is the spectral width, and *A*_*i*_ is the peak area.

1 $$ f(x)=\sum \limits_{i=1}^4\frac{A_i}{w_i\sqrt{\raisebox{1ex}{$\pi $}\!\left/ \!\raisebox{-1ex}{$2$}\right.}}\cdotp {e}^{-2{\left(\frac{x-{c}_i}{w_i}\right)}^2} $$

The four individual Gaussian functions correspond to two spin-orbit split isotope transitions. This results in tight constraints of the 12 variables in Eq. 1, enabling a robust fitting. All four central wavelength positions *c*_*i*_ are only governed by the term energies and can be treated as constant. The same applies for the spectral widths *w*_*i*_, which are primarily determined by the instrumental resolution. Since the four peaks reflect only two doublet transitions of the two isotopes, the ratio between two respective areas (e.g., *A*_1_ and *A*_2_) is constant. Namely, the spectral displacement between *c*_1_ and *c*_3_ is the isotopic shift of 15.80 pm between ^7^Li and ^6^Li [21]. The distance between *c*_1_ and *c*_2_ or *c*_3_ and *c*_4_ is the spin-orbit splitting of 15.08 pm. For calibration of the absolute spectral position, the La signal was fit to a single Gaussian function. The La peak center (*c*_*La*_) was then used to correct the Li peak center. Therefore, *A*_1_ and *A*_3_ are the only free variables. The scripts used for data preprocessing and analysis are provided in the Supplementary information (ESM1 and ESM2).

## Results

**Fig. 1:**
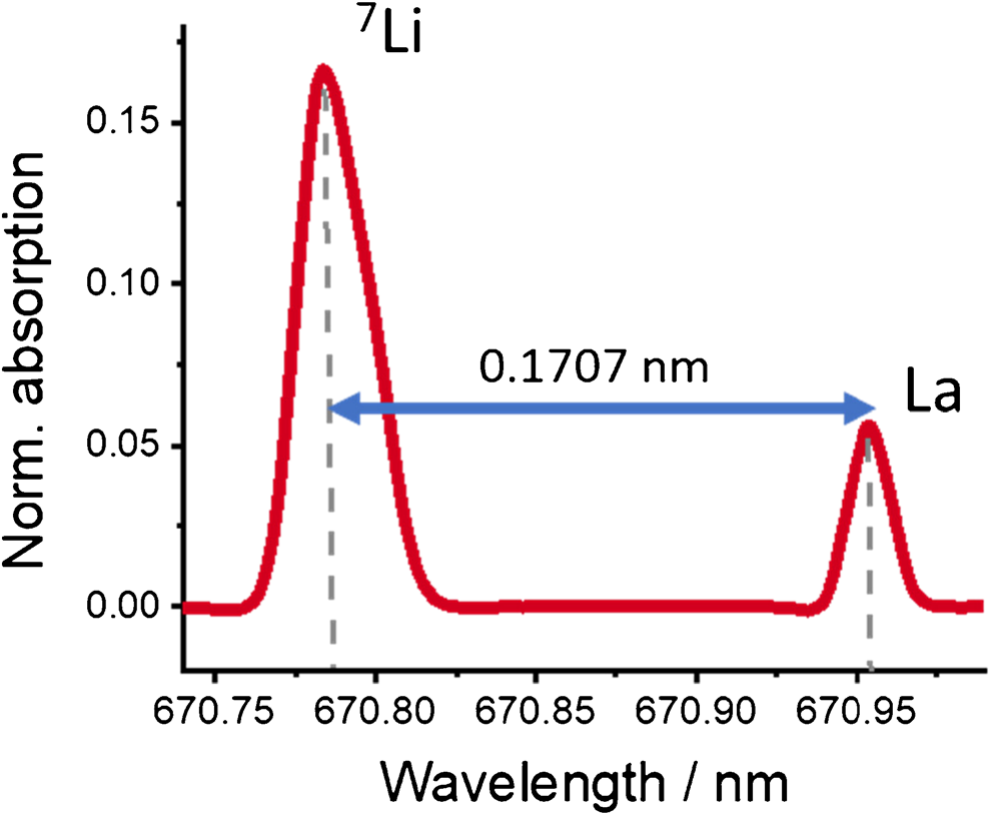
Use of La as an internal spectral standard in a ^7^Li-enriched sample

The Li characteristic mass (*m*_*c*_) which represents 1% transmittance was found to be 0.6 pg. The ID analysis was based on the isotope shift of the electronic transition 2^2^P ← 2^2^S, which exhibits two spin-orbit components for each isotope, as we previously described [21]. The nonlinear fit parameters were optimized using the spectra of ^7^Li- and ^6^Li-enriched materials. The wavelength of the spectra was corrected by using La as an internal spectral standard for the electronic transition ^4^F_5/2_ ← ^4^G°_7/2_ as shown in Fig. 1 [22].

**Fig. 2:**
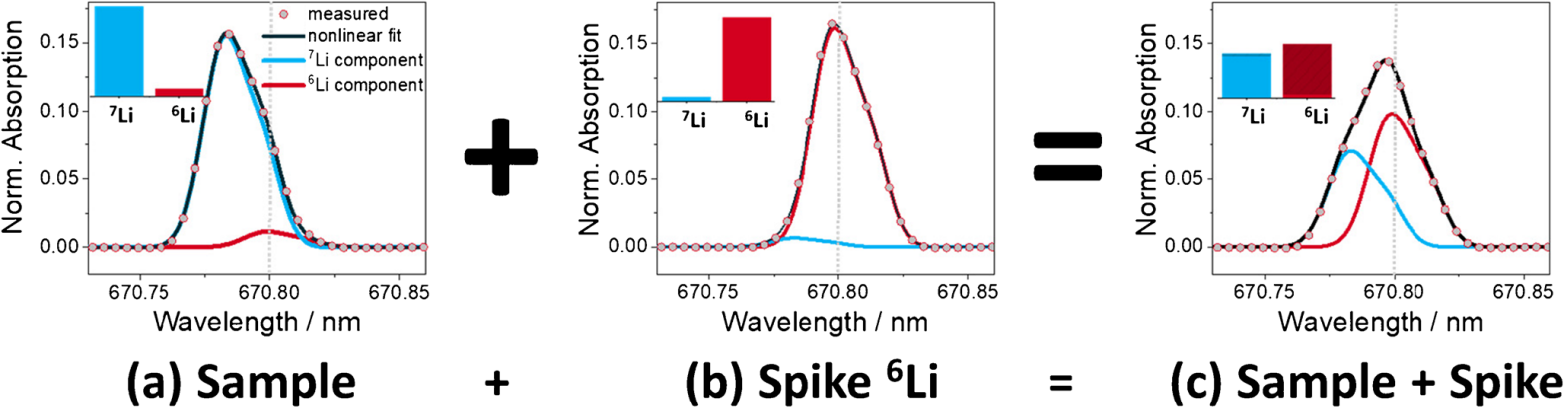
Representation of the isotope dilution high-resolution continuum source graphite furnace atomic absorption spectrometry-based approach for determination of Li by its isotope shift in the atomic spectra. (a) Spectrum of a sample with a naturally occurring isotopic composition (Li inductively coupled plasma standard) added to a ^6^Li-enriched spike (b). The combination of (a) and (b) produced the spectrum of the mixture (c). On the top left corner of each spectrum, schematic representation of the isotope amount fractions of Li is provided

The *A*_1_/*A*_2_ ratio, which is dictated by spin statistics and the Einstein coefficients of the transitions, was determined to be 2.5645 (as well as the *A*_3_/*A*_4_ ratio) and was fixed at this value. The widths *w*_1_ and *w*_3_ were determined and set to be 0.016 nm, and the widths *w*_2_ and *w*_4_ were fixed at 0.014 nm. For data analysis without correction, the peak center *c*_1_ was set at 670.7833 nm. The La-corrected Li peak center was set at *c*_1_ = *c*_*La*_ − 0.1707 nm. The ^7^Li/^6^Li isotope ratios were determined as the ratio of *A*_1_/*A*_3_. Subsequently, these ratios were fed into Eq. 2 [9] to calculate the Li contents in the serum samples, where *w*_sample_ is the Li mass fraction in the sample, *w*_spike,6Li_ is the ^6^Li mass fraction in the sample, *M*(sample) is the molar mass of Li in the sample, *M*(^6^Li) is the molar mass of ^6^Li, *x*_s*ample,*_^*6*^_*Li*_ is the amount fraction of Li in the sample, *m* is the mass of sample and added spike, and *R* represents the isotope ratio in spike, sample, and spike–sample mix, respectively. This procedure for Li quantification via ID is illustrated in Fig. 2. Natural occurring variations in the Li isotope ratios are much smaller than the uncertainties in the ID-AAS measurements. Therefore, the atomic weight interval of Li with naturally isotopic composition, i.e., between 6.9387 and 6.9438 g mol^−1^, was assumed in the serum samples [23]. The mass fraction of the spike solution was determined using reverse ID by rearranging the equation for *w*_*spike*_ and setting *w*_*spike,*_^*6*^_*Li*_ as the certified concentration of the ICP standard solution.

2 $$ {w}_{sample}={w}_{spike,{}^6 Li}\frac{M(sample)}{M\left({}^6 Li\right)\cdotp {x}_{sample,{}^6 Li}}\cdotp \frac{m_{spike}}{m_{sample}}\cdotp \frac{R_{spike}-{R}_{mix}}{R_{mix}-{R}_{sample}} $$

Table 1 summarizes the results of the Li quantification by ID-HR-CS-GFAAS. The corresponding uncertainties were calculated according to the *Guide to the Expression of Uncertainty in Measurement* supplement 1 (GUM-S1) using *GUM Workbench 2.4* (Metrodata GmbH, Germany) and *Open Monte Carlo Engine v1.2.3* (Ruediger Kessel, NIST, USA). The metrological compatibility of the ID-HR-CS-GFAAS data with the certified values of the CRMs can be evaluated by applying the *E*_n_ value, which is the difference of two values divided by the expanded uncertainty of this difference [24]. Both values are considered metrologically compatible for *E*_n_ < 1 and not metrologically compatible for *E*_n_ > 1. As shown in Table 1, the Li concentrations determined by the present ID-HR-CS-GFAAS method are metrologically compatible with the certified values.

**Table 1:** Results of isotope dilution high-resolution continuum source graphite furnace atomic absorption spectrometry (ID-HR-CS-GFAAS) and certified Li content in human serum certified reference materials (CRMs)

CRM	Certified Li content		ID-AAS without correction			ID-AAS with La correction		
	Molarity (mmol L^−1^ Li)	*U*	Molarity (mmol L^−1^ Li)	*U*	*E*_n_	Molarity (mmol L^−1^ Li)	*U*	*E*_n_
BCR 304	0.985	0.029	0.92	0.09	0.69	0.976	0.022	0.25
ERM-DA250a	0.98*	0.06*	0.94	0.09	0.37	0.983	0.018	0.05
ERM-DA251a	0.66*	0.04*	0.62	0.07	0.50	0.663	0.022	0.07
Seronorm L1	0.73	0.15	0.82	0.06	0.57	0.830	0.016	0.68
Seronorm L2	1.45	0.29	1.52	0.13	0.22	1.463	0.028	0.04

The expanded uncertainties are expressed at the 95% confidence level (*k* = 2)

*Calculated from the certified mass fraction and its density. ERM-DA250a: 1.0294 ± 0.0014 kg L^−1^, ERM-DA251a: 1.0175 ± 0.0013 kg L^−1^. Atomic weight of lithium: 6.941 ± 0.003 g mol^−1^ [25]

## Discussion

A practical approach for Li quantification in human serum is presented, which is based on the partial resolution of the isotopic shift for the electronic transition 2^2^P ← 2^2^S around the wavelength of 670.80 nm by a commercially available HR-CS-GFAAS instrument. Monitoring of this isotope shift using the La peak as a reference enables the quantification of the isotopic components with high reproducibility. Therefore, it can be used for an ID analysis by nonlinear fitting of four Gaussian curves representing the spin-orbit split doublets ^6^Li _1/2_, ^6^Li _3/2_, ^7^Li _1/2_, and ^7^Li _3/2_, respectively. The characterization of the individual transition properties (a doublet for each isotope) can be easily performed using commercially available ^6^Li- and ^7^Li-enriched materials. Although the HR-CS-GFAAS instrument provides high reproducibility for the wavelength measurements (±0.5 pixels for resolution ≈ 140,000 Δλ/λ), the accuracy is further improved by adding La as an internal spectral standard for wavelength correction. After characterization of the Gaussian parameters, only three samples need to be measured for Li quantification: (i) a ^6^Li spike, (ii) a mixture of the standard and ^6^Li spike, and (iii) a mixture of the sample and ^6^Li spike.

Additionally, the human serum samples only require acidic dilution. No matrix effects are observed, at least for the type of biological samples evaluated. The application of this procedure for Li quantification in other relevant samples having complex matrices, such as geological samples and Li-ion batteries, is under investigation. The uncertainties (*U*) obtained using the present ID-HR-CS-GFAAS method with La correction lie between 0.016 and 0.028 mmol L^−1^. The results are metrologically compatible with the certified reference material and comparable with those obtained with ID-AAS and ID-MS procedures, which afford uncertainties ranging between 0.006 and 0.015 mmol L^−1^ [15, 26, 27]. The higher-order, traceability, and commutability of the studied certified reference materials assure their representability as clinical human serum samples [28–30]. For all these, ID-HR-CS-GFAAS can be envisaged as a metrological procedure for a fast, simple, and low-cost determination of Li in human serum.

## Supplementary information

ESM 1 (TXT 1 kb)

ESM 2 (TXT 5 kb)

## Declarations

### Source of biological materials

Human serum certified reference materials were acquired from commercial providers.
